# Drug repurposing screens identify chemical entities for the development of COVID-19 interventions

**DOI:** 10.1038/s41467-021-23328-0

**Published:** 2021-06-03

**Authors:** Malina A. Bakowski, Nathan Beutler, Karen C. Wolff, Melanie G. Kirkpatrick, Emily Chen, Tu-Trinh H. Nguyen, Laura Riva, Namir Shaabani, Mara Parren, James Ricketts, Anil K. Gupta, Kastin Pan, Peiting Kuo, MacKenzie Fuller, Elijah Garcia, John R. Teijaro, Linlin Yang, Debashis Sahoo, Victor Chi, Edward Huang, Natalia Vargas, Amanda J. Roberts, Soumita Das, Pradipta Ghosh, Ashley K. Woods, Sean B. Joseph, Mitchell V. Hull, Peter G. Schultz, Dennis R. Burton, Arnab K. Chatterjee, Case W. McNamara, Thomas F. Rogers

**Affiliations:** 1 Calibr, a division of The Scripps Research Institute, La Jolla, CA USA; 2 Department of Immunology and Microbiology, The Scripps Research Institute, La Jolla, CA USA; 3 Department of Cellular and Molecular Medicine, UC San Diego, La Jolla, CA USA; 4 HUMANOID CoRE, UC San Diego, La Jolla, CA USA; 5 Department of Computer Science and Engineering, Jacobs School of Engineering, UC San Diego, La Jolla, CA USA; 6 Department of Pediatrics, UC San Diego, La Jolla, CA USA; 7 Animal Models Core Facility, The Scripps Research Institute, La Jolla, CA USA; 8 Department of Pathology, UC San Diego, La Jolla, CA USA; 9 Department of Medicine, UC San Diego, La Jolla, CA USA; 10 UC San Diego Division of Infectious Diseases and Global Public Health, UC San Diego School of Medicine, La Jolla, CA USA

**Keywords:** High-throughput screening, SARS-CoV-2, Viral infection, Infection

## Abstract

The ongoing pandemic caused by the novel severe acute respiratory syndrome coronavirus 2 (SARS-CoV-2), necessitates strategies to identify prophylactic and therapeutic drug candidates for rapid clinical deployment. Here, we describe a screening pipeline for the discovery of efficacious SARS-CoV-2 inhibitors. We screen a best-in-class drug repurposing library, ReFRAME, against two high-throughput, high-content imaging infection assays: one using HeLa cells expressing SARS-CoV-2 receptor ACE2 and the other using lung epithelial Calu-3 cells. From nearly 12,000 compounds, we identify 49 (in HeLa-ACE2) and 41 (in Calu-3) compounds capable of selectively inhibiting SARS-CoV-2 replication. Notably, most screen hits are cell-line specific, likely due to different virus entry mechanisms or host cell-specific sensitivities to modulators. Among these promising hits, the antivirals nelfinavir and the parent of prodrug MK-4482 possess desirable in vitro activity, pharmacokinetic and human safety profiles, and both reduce SARS-CoV-2 replication in an orthogonal human differentiated primary cell model. Furthermore, MK-4482 effectively blocks SARS-CoV-2 infection in a hamster model. Overall, we identify direct-acting antivirals as the most promising compounds for drug repurposing, additional compounds that may have value in combination therapies, and tool compounds for identification of viral host cell targets.

## Abstract (web summary)

Here, the authors perform repurposing screens of the ReFRAME drug library in two cell lines and identify inhibitors of SARS-CoV-2 infection. Antiviral activity of prodrug MK-4482 is confirmed in hamsters.

## Introduction

In early December of 2019, the severe acute respiratory syndrome coronavirus 2 (SARS-CoV-2) was identified as the cause of rapidly increasing numbers of severe pneumonia-like symptoms termed COVID-19^1^. Since then, SARS-CoV-2 has rightfully been given its pandemic status by the World Health Organization (WHO). As of 10 February 2021 SARS-CoV-2 has spread throughout the world causing more than 106,555,200 confirmed infections and more than 2,333,440 reported deaths in 223 different countries^2^. Development of several effective anti-SARS-CoV-2 vaccines will no doubt contribute to the control of the pandemic, however emergence of SARS-CoV-2 strains with escape mutations that render some of the vaccines less effective and overall limited global supply of COVID-19 vaccines make a case for continued effort to identify therapeutic interventions. Yet, despite an extensive effort by the research community, antiviral treatment options for COVID-19 remain limited. These include corticosteroids such as dexamethasone^3^ and the intravenously delivered antiviral remdesivir^4–6^ for treatment of patients with severe or critical COVID-19. Remdesivir, a nucleotide analog prodrug and an RdRp inhibitor with broad antiviral activity demonstrated positive clinical endpoints in a Phase III Adaptive COVID-19 Treatment Trial (median time to recovery shortened from 15 to 11 days)^7^ that justified its emergency use authorization by the US Food & Drug Administration for treatment of hospitalized COVID-19 patients^8^. However, it, together with hydroxychloroquine, lopinavir and interferon regimens has recently failed to reduce mortality of hospitalized COVID-19 patients in a large multi-center WHO SOLIDARITY trial^9^. Remdesivir’s modest efficacy and intravenous delivery make the discovery of new or supplemental therapies that produce greater clinical improvements and can be administered outside of a hospital setting (i.e. orally) highly desirable. The Repurposing, Focused Rescue, and Accelerated Medchem (ReFRAME) drug collection is a drug repurposing library containing nearly 12,000 small-molecule drugs shown to be appropriate for direct use in humans^10^ and provides a rich resource to discover treatments that may be used as monotherapies or in combination with remdesivir to further enhance treatment efficacy and tolerability. Here we report results from ReFRAME screens in two different cell-based SARS-CoV-2 infection assays and in a remdesivir potentiation format, and the profiling of the identified hits in secondary orthogonal assays. This screening cascade and subsequent hit prioritization identified and validated our most promising hit, MK-4482, as a potent inhibitor of SARS-CoV-2, in vitro findings which translated to an in vivo hamster model of SARS-CoV-2 infection. Other hits identified in these studies have the potential for repurposing following further evaluation in advanced models or may serve as tool compounds in elucidation of coronavirus replication pathways.

## Results

### High-throughput HeLa-ACE2 ReFRAME screen against SARS-CoV-2

**Fig. 1:**
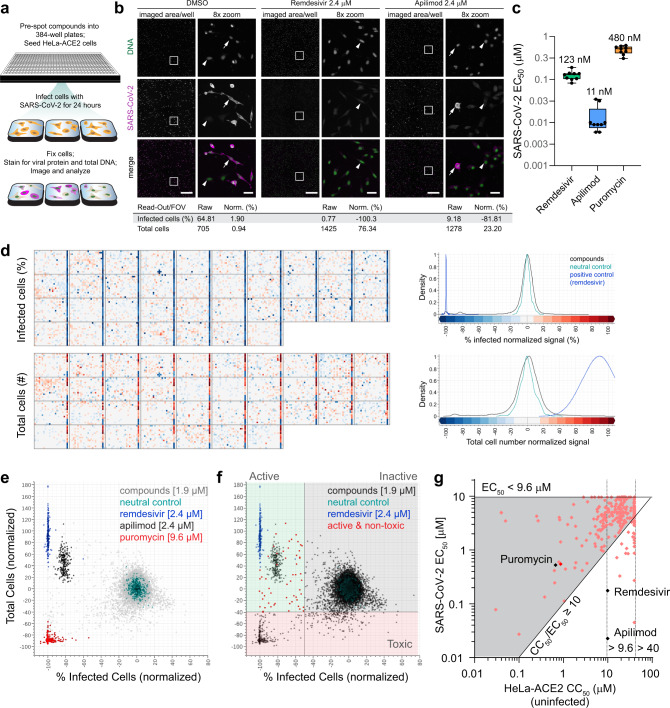
A primary cell-based HCI assay identifies compounds active against SARS-CoV-2 infection. **a** Simplified assay workflow. **b** Representative images from dimethyl sulfoxide (DMSO)-, remdesivir- or apilimod-treated wells. The entire imaged area per well (four fields of view taken with a ×10 objective and stitched together) is shown for each treatment, as well as an 8-fold magnified segment demarcated with a white box. DNA signal [4′,6-diamidino-2-phenylindole (DAPI)] is colored green, and the virus visualized with immunofluorescence is colored magenta. Infected (arrow) and uninfected (arrowhead) cells are indicated; 500 µm and 50 µm scale bars are shown in the composite and magnified images, respectively. Raw and normalized (Norm.) values calculated from the images are shown. **c** Box and whiskers plot of SARS-CoV-2 assay control EC_50_s obtained from *n* = 9 independent biological experiments with all data points shown. Whiskers indicate minimums and maximums, the box extends from the 25th to 75th percentile, the center of the box is the median and geomeans are reported. **d** Heat map images of normalized data from 1.9 µM ReFRAME screening plates. Normalized activity values for percentage infected cells and total cell numbers are indicated according to the scale bar and density plot for compound and control wells is shown. DMSO-treated wells are in column 24 and positive control-treated wells (blocks of wells with 2.4 µM remdesivir, 2.4 µM apilimod, or 9.6 µM puromycin) in column 23. Density plots representing the frequency of values associated with each well type are shown on the right. **e** Distribution of 1.9 µM ReFRAME screen data for compound and control wells. **f** Screen hit selection thresholds. **g** Library dose-response reconfirmation results, with the SARS-CoV-2 EC_50_ of each compound plotted against its host-cell toxicity CC_50_ as assessed in uninfected HeLa-ACE2 cells. Gray area defines non-selective compounds, where the selectivity index (CC_50_/EC_50_) is < 10. Dotted lines represent maximal concentrations tested in dose-response studies for the assay compounds (40 µM) and controls apilimod and remdesivir (9.6 µM). Activities of controls (black diamonds) and assay compounds (pink diamonds) are shown. Activity of the ReFRAME library copy of puromycin that was screened as part of this hit reconfirmation is also indicated (red diamond). Source data are provided as a Source Data file.

To identify compounds that could inhibit replication of SARS-CoV-2 in human cells, we developed a high-content imaging (HCI) 384-well format assay using HeLa cells expressing the human SARS-CoV-2 receptor, the angiotensin-converting enzyme 2, or ACE2 (HeLa-ACE2). In this assay HeLa-ACE2 cells are infected with SARS-CoV-2 virus in the presence of compounds of interest and infection is quantified 24 h later (Fig. 1a). The assay relies on immunofluorescent detection of SARS-CoV-2 proteins with plasma purified from patients exposed to the virus, which together with host-cell nuclear staining allows for quantification of the percent infected cells in each well (Fig. 1b).

We validated the assay using compounds with reported activity against SARS-CoV-2: remdesivir (GS-5734)^11^ (half maximal effective concentration (EC_50_) = 123 nM $\times \div$ 1.276; geometric mean $\times \div$ geometric standard deviation of nine independent experiments, *n* = 9, see Methods section) and the PIKfyve inhibitor apilimod (EC_50_ = 11.3 nM $\times \div$ 1.910, *n* = 9; Fig. 1b). Remdesivir at elevated concentrations (2.4 µM) was able to nearly eliminate all detectable infection within cells (Fig. 1c) and was a positive control, with data normalized to it and neutral DMSO control wells. While apilimod was more potent than remdesivir, it had a fractionally lower maximal efficacy (85−90% of uninfected cells at the highest effective concentrations) compared to remdesivir. We also assessed compound toxicity by quantifying the total cell numbers per well in the context of infection and in uninfected cells, with cytotoxic protein synthesis inhibitor puromycin as a positive control in both assays (cell count in infection assay EC_50_ = 1,552 nM $\times \div$ 1.257, *n* = 9; uninfected HeLa-ACE2 half maximal cytotoxic concentration (CC_50_) = 658 nM $\times \div$ 1.302, *n* = 9). Notably, a concomitant increase in cell numbers coincided with the antiviral activity of remdesivir and apilimod, likely by counteracting a modest cytopathic effect caused by infection in HeLa-ACE2 cells (Fig. 1b–e). Altering the multiplicity of infection had limited effects on the potency of control compounds in the same experiment, with a 2.7-fold increase in remdesivir’s EC_50_ from MOI = 0.09 to MOI = 2.2, and a 3.7-fold increase in apilimod’s EC_50_, but not that of puromycin’s (Supplementary Fig. 1).

Using the developed assay, we ran a pilot screen to assess the activity of 148 small molecules with suspected therapeutic potential against coronavirus infections mined from the available literature^12^ (Robust Z′ factor (RZ′), a measure of assay quality, of 0.84). We identified 19 compounds with an EC_50_ < 9.6 µM and, based on data obtained from an uninfected HeLa-ACE2 24 h live/dead assay, 16 of these were selective (uninfected HeLa-ACE2 CC_50_/SARS-CoV-2 EC_50_ > 10 or uninfected HeLa-ACE2 CC_50_ > 40 µM; Supplementary Data 1). This included library/screening lots of apilimod and remdesivir that were “rediscovered” in the assay.

Next, we screened the 12,000-compound ReFRAME repurposing library at a final concentration of 1.9 µM and 9.6 µM. HeLa-ACE2 assay quality was maintained throughout both screens (RZ′ of 0.87 and 0.72, respectively; Table 1) and a clear distinction was apparent in the activity profiles of DMSO vehicle- (neutral control), remdesivir- (positive control), apilimod-, and puromycin- (toxicity control)-treated wells (Fig. 1d, e). Hits were selected based on demonstration of > 50% reduction in the number of infected cells per well (< −50% activity normalized to neutral controls minus inhibitors) and < 40% toxicity based on the total cell number per well (> −40% activity normalized to compound activities, including 9.6 µM puromycin; Fig. 1e, f) identifying 61 primary hits at 1.9 µM and 266 primary hits at 9.6 µM screening concentrations (hit rates of 0.51 and 2.24%, respectively), with a total of 311 hits.

**Table 1:** ReFRAME primary and validation screen statistics for HeLa-ACE2 and Calu-3 SARS-CoV-2 infection assays.

Assay	HeLa-ACE2/SARS-CoV-2			Calu-3/SARS-CoV-2
ReFRAME library screening concentration	[1.9 µM]	[9.6 µM]	Total^b^	[2.5 µM]
Compounds screened	11,861	11,861	11,861	11,861
Primary hits^a^	61	266	311	235
Average RZ′	0.8695	0.7241	0.7968	0.744
Hit rate (%)	0.51	2.24	2.75	1.98
Tested in primary dose response (DR)	60	265	310	220
EC_50_ < 10 µM	43	197	225	145
Reconfirmation rate (%)	71.7	74.3	72.6	65.9
EC_50_ < 10 µM, CC_50_/EC_50_ > 10	15	53	58	42
Potent and selective primary hits (%)	25.0	20.0	18.7	29.0
Hits tested as fresh powders	–	–	75^c^	88
EC_50_ < 10 µM	–	–	73	87
EC_50_ < 10 µM, SI (CC_50_/EC_50_) > 10 or CC_50_ > max	–	–	49	41
Potent and selective reconfirmed hits (%)	–	–	65.3	46.6

^a^Primary hit thresholds in HeLa-ACE2: > 50% inhibition of infection, < 40% cell toxicity; 6 border-line hits included in 1.9 μM; in Calu-3: > 50% inhibition of infection, < 80% cell toxicity; 25 border-line hits included with > 40% inhibition of infection and > 40% increase in cell count (protection from virus-induced cell death).

^b^Non-overlapping hits from 1.9 and 9.6 μM HeLa-ACE2 screens.

^c^58 ReFRAME hits and 17 non-overlapping hits from pilot.

To reconfirm and assess potency and selectivity of the primary hits we tested 310 of the available compounds in a 10-point, 1:3 dilution, dose-response format with a top concentration of 9.6 µM. Of these, 225 (72.6%) demonstrated activity with EC_50_ < 9.6 µM against SARS-CoV-2. However, many of the primary screen hits were also cytotoxic, with an unacceptably low selectivity ratio as determined in uninfected HeLa-ACE2 cells (uninfected CC_50_/SARS-CoV-2 EC_50_ < 10; Table 1 and Fig. 1g). Because viruses rely on host machinery for replication, it was not unexpected that many of the compounds with antiviral activity also affected vital host processes. Between the small pilot and the ReFRAME screen we identified 75 potent (EC_50_ < 9.6 µM) and selective (CC_50_/EC_50_ > 10 or CC_50_ > 39.8 µM) compounds and of these, 73 reconfirmed as potent and 49 reconfirmed as potent and selective in HeLa-ACE2 cells when fresh powder stocks were tested (Table 1).

### HeLa-ACE2 remdesivir potentiation screen and synergy studies

Combination therapies have the potential to increase efficacy of treatment while reducing drug dose of either or both combinations partners, and thus prevent side effects that may be associated with administration of higher doses. Drug combinations can also slow the acquisition of drug resistance. Drug synergy, the increase in activity of the combination therapy beyond what is expected of an additive interaction is rare, yet additive effects themselves have the potential to improve therapy regimens.

To identify compounds that interact synergistically with the FDA-approved COVID-19 drug remdesivir, we carried out a second unbiased ReFRAME screen in the presence of low concentrations (80 nM) of remdesivir. The activity of hits from this screen were assessed in the presence and absence of remdesivir. Based on a perceived shift in activity in the presence of remdesivir, compounds were tested in a checkerboard 10 × 10 synergy matrix and results analyzed using the *synergyfinder* package^13^ in R to assess the interactions between the tested compounds using the Zero Interaction Potency Model (ZIP)^14^ (see Methods section). From this screen we identified the nucleoside analog riboprine (N6-isopentenyladenosine, previously investigated as an antineoplastic agent, for treatment of nausea and surgical site infections, and a component of CitraNatal 90 DHA, a prescription prenatal/postnatal multivitamin/mineral tablet) and a folate antagonist 10-deazaaminopterin (an antineoplastic compound currently in Phase II stage of development) as having activities that synergized with those of remdesivir. The synergistic effects for both compounds were observed across specific concentrations, signified as peaks within the 3-dimensional synergy score landscape (Supplementary Fig. 2a, b), prompting closer scrutiny of activity of each compound.

Riboprine achieved maximal (100%) efficacy over the range of concentrations tested, but addition of EC_2_ of remdesivir shifted its EC_50_ from 12 µM to 3.6 µM, and addition of EC_24_ of remdesivir increased its potency further to EC_50_ = 1.6 µM (Supplementary Fig. 2a). 10-deazaaminopterin showed only 40% maximal efficacy over the range of concentrations tested, but the addition of EC_2_ of remdesivir caused an increase of maximal efficacy from 40% to nearly 65% (where a shift of 2% would be expected) and addition of EC_24_ of remdesivir increased maximal efficacy of the combination from 40% to > 80% (Supplementary Fig. 2b). The mechanism of action behind the observed synergies remains to be determined. Riboprine has been reported to block uridine and cytidine import^15^ and through inhibition of protein prenylation to inhibit autophagy^16^ which could impact RNA catabolism^17^ whereas 10-deazaaminopterin has been suggested to inhibit folate-dependent enzymes of the purine biosynthesis pathway^15–18^. Therefore, treatment with either riboprine or 10-deazaminopterin may result in reduced intracellular nucleoside pools and in this way synergize with RdRp inhibition by the adenosine nucleoside analog remdesivir. While these findings indicate a promising avenue for further investigation of combination therapies for treatment of COVID-19, the adverse effects of these agents (e.g., inhibition of immunity by 10-deazaaminopterin) would need to be carefully considered in the design and dose selection of in vivo validation experiments.

In addition to an unbiased screening approach, we also performed synergy interaction studies comparing full dose response of remdesivir against the dose responses of 19 HeLa-ACE2 ReFRAME hits with attractive safety and pharmacokinetic profiles (Supplementary Data 1). We found no synergy between remdesivir and the compounds tested; however, the combinations were additive, including that of nelfinavir with remdesivir (Supplementary Fig. 2c, Table 1, and Supplementary Data 1), suggesting these drugs, if they were to prove efficacious in vivo, could potentially be co-administered with remdesivir to increase the overall safety and efficacy of treatment, while limiting the evolution of drug resistance.

### High-throughput Calu-3 ReFRAME screen against SARS-CoV-2

To complement the relatively rapid 24 h HeLa-ACE2 assay and prioritize hits, we developed a second, more physiologically relevant infection assay using Calu-3 cells that relied on the same antibody detection and a similar assay workflow, with a readout at 48 h post SARS-CoV-2 infection (hpi; see Methods section). Calu-3 are human lung epithelial cells that endogenously express both the ACE2 receptor and the host serine protease TMPRSS2, which is required for SARS-CoV-2 Spike protein processing and viral entry into host cells^19^, while the robust infection in HeLa-ACE2 cells, which lack TMPRSS2 expression, is likely dependent on endosomal, cathepsin-mediated viral entry pathway that has been a generally recognized mechanism for coronaviruses^20^. Remdesivir was active in Calu-3 cells (EC_50_ = 444 nM ×÷ 1.514 (*n* = 4)) (Supplementary Data 1), as was the TMPRSS2 inhibitor nafamostat mesylate (EC_50_ = 24 nM ×÷ 1.55 (*n* = 3)) (Supplementary Data 2). In contrast to the HeLa-ACE2 assay, cytopathic effect was more pronounced in the Calu-3 assay (likely due to higher MOI and longer incubation times used). As a result, antiviral compounds also protected the Calu-3 cells from virus-induced cell death, providing a second metric related to compound antiviral activity.

**Fig. 2:**
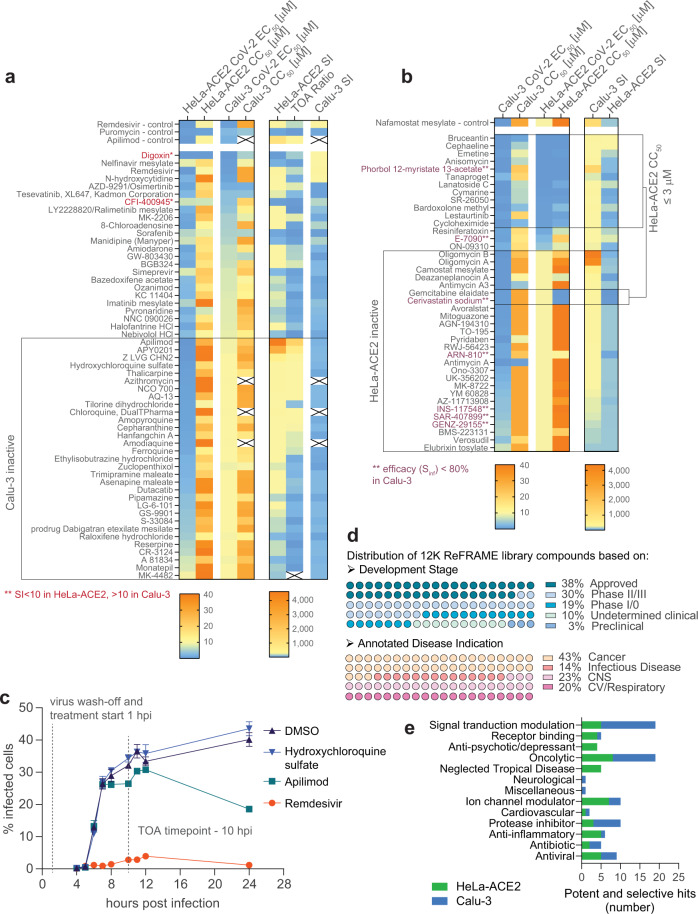
HeLa-ACE2 and Calu-3 cell-based assays identify largely distinct sets of potent and selective compounds with anti-SARS-CoV-2 activity. **a** Heat map of activities of HeLa-ACE2 ReFRAME hits reconfirmed with fresh powder stocks in HeLa-ACE2 and Calu-3 cells (EC_50_, CC_50_, selectivity index (SI)) and the time of addition (TOA) vs standard 24 h assay HeLa-ACE2 antiviral EC_50_ ratio. Double asterisk indicates two compounds identified in primary screening that were not selective in powder reconfirmation in HeLa-ACE2 cells but were active and selective in Calu-3 cells. X indicates missing data. **b** Heat map of activities of Calu-3 ReFRAME hits reconfirmed with fresh powder stocks in Calu-3 and HeLa-ACE2 cells (EC_50_, CC_50_, SI). Double asterisk indicates compounds with antiviral efficacy in Calu-3 < 80%. All raw data from experiments graphically represented in **a** and **b** are reported as part of Supplementary Data 1 and 2 and include the independent number each measurement was performed (≥ 3 for most compounds/assays). **c** HeLa-ACE2 TOA time course showing SARS-CoV-2 % infected cells at different times post-infection. Cells were infected with virus for one hour, washed extensively prior to compound treatment, and fixed at the indicates times. The 10 hpi timepoint was chosen for the TOA assay. Means ± s.d. are shown, *n* = 3 technical replicates examined over one independent experiment. **d** The composition of the ReFRAME repurposing library with respect to clinical stage of development and disease indication. **e** Classification of HeLa-ACE2 and Calu-3 potent and selective hits according to their functional annotation. Source data are provided as a Source Data file.

Notably, we found that the majority of the 52 HeLa-ACE2 pilot and ReFRAME hits (49 hits and 3 compounds of interest: Digoxin, CFI-400945, MK-4482) were either not active (58%, 30/52) or not selective in the Calu-3 cell-based assay (Fig. 2a and Supplementary Data 1). This limited overlap in activities in HeLa-ACE2 and Calu-3 cells prompted us to re-screen the ReFRAME library using Calu-3 cells. The screen was carried out at a final concentration of 2.5 µM, RZ′ = 0.744, and we identified 235 primary hits that demonstrated > 50% inhibition of infection, < 80% cell toxicity or > 40% inhibition of infection and > 40% increase in cell count (protection from virus-induced cell death). Of these, 145 were moderately active when tested in a dose-response format (EC_50_ < 10 µM), but only 42 were also selective (CC_50_/EC_50_ > 10 or CC_50_ > 30 µM). We chose 88 of putative hit compounds to test as fresh powder stocks (CC_50_/EC_50_ > 5 or CC_50_ > 30 µM, CC_50_/EC_50_ < 5 but with less than a 50% reduction in uninfected cytotoxicity assay, and 3 extra compounds with EC_50_ < 1 µM showing protection in infected cell count readout) and 87 reconfirmed as potent and 41 reconfirmed as also selective in Calu-3 cells (Table 1). The 41 reconfirmed Calu-3 ReFRAME hits were likewise re-tested in the HeLa-ACE2 infection assay. Of these, 63% (26/41) were inactive against SARS-CoV-2 in HeLa-ACE2 cells, whereas 34% (14/41) were active but strongly cytotoxic, with a CC_50_ < 3 µM in uninfected HeLa-ACE2 cells (Fig. 2b and Supplementary Data 2).

### Identifying endosomal cathepsin-mediated entry inhibitors

As one likely source of limited activity of HeLa-ACE2 ReFRAME hits in the Calu-3 assay is the entry mechanism used by the virus in each cell line, we established a time of addition (TOA) assay in HeLa-ACE2 cells to identify cathepsin-mediated viral entry inhibitors among the ReFRAME hits, which are unlikely to be active in the context of TMPRSS2-entry. To first determine kinetics of infection, HeLa-ACE2 cells were infected for 1 h with SARS-CoV-2, after which un-adsorbed virus was washed off, and cells plated in 384-well plates in the presence of DMSO, hydroxychloroquine, apilimod, or remdesivir at a final concentration of 10 µM. Cells in wells were fixed as indicated, from 4 to 24 hpi and percent infected cells at each timepoint were quantified (Fig. 2c). In all treatments except for remdesivir, viral infection was first apparent by antibody staining at 6 hpi and reached near maximal levels at 10 to 12 hpi. Loss of activity of both apilimod and hydroxychloroquine when treatment was initiated at 1 hpi indicates that these compounds block viral entry in HeLa-ACE2 cells while remdesivir treatment effectively blocked the infection, despite the initiation of treatment at 1 hpi, in line with its direct antiviral mechanism of action.

Based on these results, we used the 10 hpi timepoint to limit cycles of replication in a TOA assay in which we assessed the activity of all HeLa-ACE2 hits in dose response. We found that 33% (10/30) of compounds which were inactive in Calu-3 cells were entry inhibitors in HeLa-ACE2 based on the reduction of their activity in the TOA assay, i.e., an EC_50_ ratio of > 10 between the standard 24 h and the TOA assay (Fig. 2a, Supplementary Data 1). In contrast, no compound that was also active in Calu-3 cells (EC_50_ < 10 µM) could as clearly be classified as an entry inhibitor at that threshold. Osimertinib and MK-2206 each had a ratio > 8, suggesting they may be involved in viral entry in HeLa-ACE2 cells, however their antiviral activity in Calu-3 cells was unspecific (SI < 2).

### ReFRAME hit prioritization and validation

**Fig. 3:**
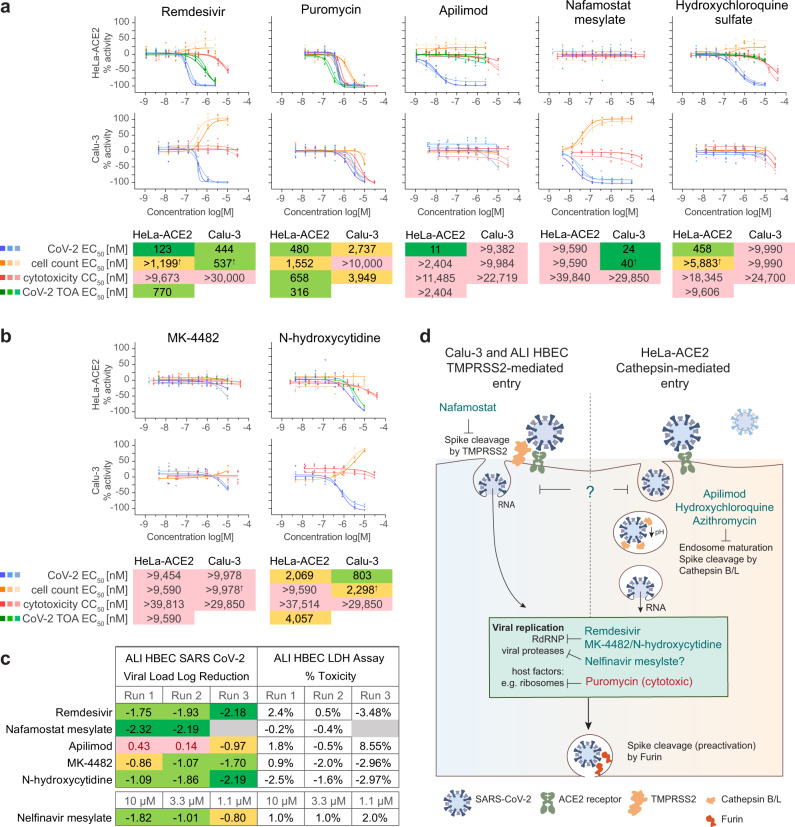
Direct acting antiviral compounds are active in multiple cell-based infection assays. **a** Representative dose-response curves of assay controls (**a**) and MK-4482 prodrug and its parent, N-hydroxycytidine (**b**) from three or two independent experiments in HeLa-ACE2 and Calu-3 assays. Individual values of technical triplicates, compound structures, and geomeans for each infection assay (SARS-CoV-2 EC_50_ and total cell count EC_50_) and uninfected cytotoxicity CC_50_ are shown. HeLa-ACE2 TOA EC_50_ geomeans are also shown as applicable. EC_50_s or CC_50_s < 200 nM are marked with dark green background, < 1000 nM with light green, and < 9000 nM with yellow. Inactive compounds or with EC_50_s or CC_50_s > 9000 nM are marked with pink. ^↑^ indicates an EC_50_ for an increasing dose-response curve slope. **c** Viral log reduction in apical supernatants determined with RT-qPCR and percentage toxicity determined with an LDH assay in ALI HBEC SARS-CoV-2 infection model following a 3-day treatment with 5 µM of each indicated compound. Dark green cell background indicates a > 2-fold log reduction compared to neutral controls, light green indicates a > 1-fold log reduction, yellow a < 1-fold log reduction, and pink indicates a viral load increase. **d** Diagram illustrating differences between compound activities observed in different cell infection models as related to different entry mechanisms available in each cell line. Source data are provided as a Source Data file.

The ReFRAME library is a collection of bioactive small molecules, many of which are approved drugs or in clinical phases of development and used for a wide assortment of indications (Fig. 3d). The top five classes of potent and selective compounds reconfirmed as powders in the HeLa-ACE2 screen were oncolytic compounds (9), ion channel modulators (7), anti-inflammatory (5), antiviral (5) and signal transduction modulators (5), whereas in the Calu-3 screen the top five classes were signal transduction modulators (14), oncolytic compounds (11), protease inhibitors (7), antibiotics (3), and ion channel modulators (3) (Fig. 3e). A fifth of the potent and selective hits in both screens could be classified as oncolytic drugs, reflecting the reliance of the virus on host-cell processes present in rapidly proliferating cells. The identification of compounds belonging to anti-psychotic and anti-parasitic (neglected tropical diseases) classes exclusively in HeLa-ACE2 cells may reflect the cationic amphiphilic nature of some of these molecules and their ability to accumulate in and impact acidic intracellular compartments (e.g., late endosomes/lysosomes). Resultant dysregulation of the endo-lysosomal pathway and lipid homeostasis has been suggested to impair viral entry and/or replication^21^ and this mode of action has been speculated for amiodarone and hydroxychloroquine, both identified as potent and selective hits against SARS-CoV-2 in the HeLa-ACE2 screen (Fig. 2a and Supplementary Data 1). However, only hydroxychloroquine was identified as an entry inhibitor in our assay. Conversely, an enrichment for protease inhibitors active in the Calu-3 assay, may reflect their inhibition of the TMPRSS2-mediated entry pathway in those cells.

From compounds identified as hits in our primary screens of high interest were compounds with a profile like that of remdesivir (Figs. 2a, b and 3a), which were active and selective in both HeLa-ACE2 and Calu-3 assays and were not classified as entry inhibitors in HeLa-ACE2 cells. The parent of prodrug MK-4482, N-hydroxycytidine matched that profile, although MK-4482 was itself not active in vitro, likely due to lack of metabolism that would turn it over to its active form (Figs. 2a and 3b). Additionally, compounds such as nafamostat mesylate, the TMPRSS2 inhibitor, active in Calu-3 but not active in HeLa-ACE2 cells (Figs. 2b and 3a) had the potential to be active in advanced models of infection. Conversely, entry inhibitors in HeLa-ACE2 cells that are not active in Calu-3 cells (e.g., apilimod, hydroxychloroquine, and azithromycin; Figs. 2a and 3a) were deprioritized. It is currently unclear whether compounds that block cathepsin-mediated entry of SARS-CoV-2 have any potential to improve disease outcomes, and if cathepsin-mediated entry by SARS-CoV-2 observed in vitro is relevant for its pathogenesis (e.g., dissemination of the virus to sites within the body that do not express TMPRSS2). However, failure of hydroxychloroquine to meet clinical endpoints in treating COVID-19^22^ suggests these compounds may not be effective as front-line therapies. Finally, it is possible that certain compounds are active in only one cell line due to metabolism or other cell-specific properties, and further testing is required to determine their suitability for downstream drug development.

Based on our prioritization, we tested activity of representative hits against SARS-CoV-2 in an orthogonal air–liquid interface primary human bronchial epithelial cell (ALI-HBEC) model of infection. As expected, remdesivir and nafamostat mesylate inhibited viral replication in ALI-HBECs, while apilimod did not. Furthermore, nelfinavir mesylate, MK-4482 and its parent *N*-hydroxycytidine all caused a > 1-log reduction in apical viral loads at 72 hpi (Fig. 3c). These results agreed with our model of hit prioritization (Fig. 3d).

Overall, we identified approved oral drugs halofantrine HCl, nelfinavir mesylate, simeprevir, and manidipine as hits of highest interest due to their activity in both assays and their relatively high exposures or a long history of use as therapeutic agents and therefore potential to be quickly repurposed as COVID-19 treatments following further efficacy vetting in animal models. The viral protease inhibitors nelfinavir and simeprevir have reported good plasma exposures and based on their described mode of action they may inhibit SARS-CoV-2 directly. The approved calcium-channel blocker manidipine has low plasma exposure but may have the potential to improve COVID-19 disease outcomes for patients. Nine other compounds in various stages of development also have high likelihood to show efficacy due to their potency in the screening assays or pharmacokinetic profiles (Table 2). TO-195 and RWJ-56423 are both trypsin inhibitors and avoralstat is a kallikrein inhibitor active in Calu-3 cells which may block viral entry. The p38 mitogen-activated protein kinase (MAPK) inhibitor, LY222820/Ralimetinib mesylate, was active in both HeLa-ACE2 and Calu-3 assays and was previously shown to inhibit replication of other coronaviruses via inhibition of p38 MAPK^23^. Thus, p38 MAPK may be an important host target for inhibiting coronavirus replication. Of note, *N*-hydroxycytidine, the parent of the prodrug MK-4482 (Molnupiravir, EIDD-2801) was a potent and selective hit in both the HeLa-ACE2 and Calu-3 assays (Fig. 3b). MK-4482 is an oral antiviral nucleoside analog previously shown to be effective in ferrets against influenza^24^, to inhibit multiple coronaviruses in human airway epithelial cells and SARS-CoV infection in mice^25^, to block SARS-CoV-2 transmission when administered therapeutically in ferrets^26^, and in a humanized mouse model^27^. It is currently being evaluated by Ridgeback Biotherapeutics and Merck in treatment of COVID-19 patients.

**Table 2:** Attractive reconfirmed hits with activity and selectivity against SARS-CoV-2.

Compound/drug name	Target/mechanism	Clinical stage	PK exposure			HeLa-ACE2			Calu-3	
			Approx. *C*_max_	Approx. *t*_1/2_ (hours)	Refs.	SARS-CoV-2 EC_50_ (µM)	Uninf. cells CC_50_ (µM)	Synergy score with remdesivir (δ)	SARS-CoV-2 EC50 (µM)	Uninf. cells CC50 (µM)
Remdesivir (GS--5734)	RdRP inhibitor	Emergency FDA registration	5 µM (human oral)	1	^33^	0.127 (0.123)*	9.97 (9.67)*	n/a	0.606 (0.444)*	> 30 (> 30)*
Halofantrine HCl	Antimalarial; hemozoin inhibitor	Registered	1 µM (human oral)	58	^34,35^	0.33	18.71	−3.51	> 8.470	> 22.59
Manidipine	Ca^2+^-channel blocker related to amlodipine	Registered	10 nM (human, oral)	3	^36,37^	6.89	> 17.07	−4.73	5.481	> 30
Nelfinavir mesylate	HIV protease inhibitor	Registered	17 µM (human oral)	7	^38,39^	> 8.643	> 18.02	−1.23	0.361	> 9.40
Simeprevir	Hepatitis C NS3/4A protease inhibitor	Registered	32 µM (human oral)	16	^40,41^	7.88	> 22.17	−0.67	7.704	> 22.39
Avoralstat	Plasma kallikrein (KLKB1) inhibitor	Phase III	364 nM (human, oral)	14	^42^	> 9.6	> 40	n/d	0.387	> 30
Bardoxolone methyl	Antiinflammatory; NFkB inhibitor; Nrf2 activator	Phase III	50 nM (human, oral)	39	^43^	0.541	3.1	n/d	0.079	3.06
Mitoguazone	Polyamine biosynthesis inhibitor	Phase III	120 µM (human, IV)	175	^44^	> 9.6	> 40	n/d	0.446	> 30
N4-hydroxycytidine	RdRP inhibitor (MK-4482 parent)	Phase II/III	3.3 µM (NHP, oral)	1	^45^	2.069	> 37.514	n/d	0.803	> 29.85
Ono-3307	Anticoagulant	Phase II	n/d	n/d	^46,47^	> 9.6	> 40	n/d	1.602	> 30
Ralimetinib mesylate/LY2228820	MAPK p38 inhibitor	Phase II	5 µM (human, oral)	190	^48,49^	1.874	27.36	n/d	3.829	13.77
8-Chloroadenosine	Telomerase reverse transcriptase (TERT) inhibitor	Phase I/II	n/d	n/d	–	0.927	> 11.86	n/d	4.94	> 27.75
TO-195	Trypsin inhibitor	Phase I	n/d	n/d	–	> 6.4	> 40	n/d	0.657	> 30
RWJ-56423	Trypsin inhibitor	Clinical	n/d	n/d	–	> 9.6	> 40	n/d	1.228	> 30

*n/d* determined

*indicates activities for remdesivir control

### MK-4482 oral dosing is protective against SARS-CoV-2-infection

**Fig. 4:**
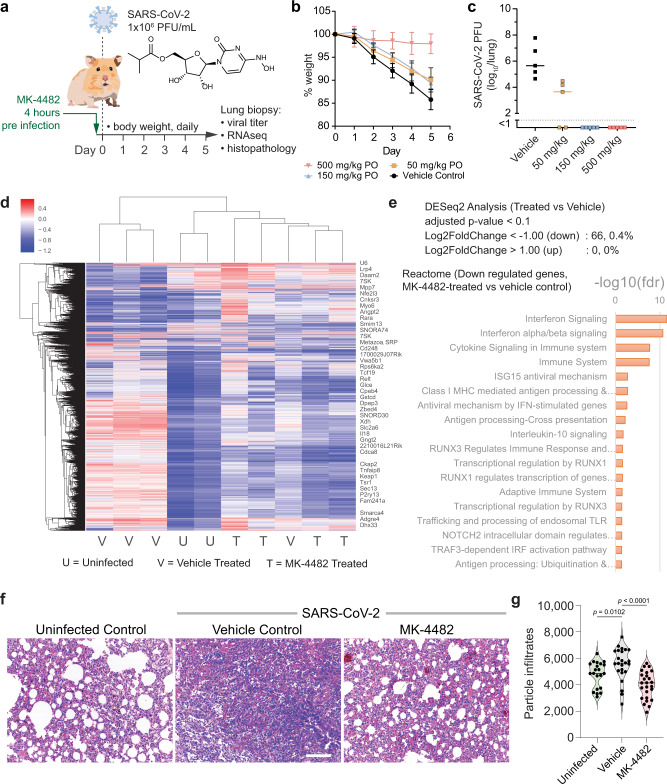
Orally administered MK-4482 blocks SARS-CoV-2 infection in Golden Syrian hamster model of infection. **a** Compounds were administered at indicated doses to Golden Syrian hamsters 4 h prior to intranasal infection with SARS-CoV-2. Hamsters were weighed each day and at day 5 were sacrificed and lung biopsies were performed. **b** Hamster weight following MK-4482 treatment and SARS-CoV-2 infection. Percent weight was calculated from day 0 for all animals. Mean weights ±s.d. are shown, *n* = 5 animals per group examined over one independent experiment. **c** SARS-CoV-2 viral load as assessed by plaque quantification from homogenized lung tissue at day 5 after infection. Medians and individual values are shown, *n* = 5 animals per group examined over one independent experiment. **d** Heatmap and hierarchical clustering of uninfected (U), vehicle-treated (V), and MK-4482-treated (500 mg/kg) (T) hamster lung samples. Genes were selected in an unbiased manner by using high values of the mean absolute deviation after applying StepMiner twice to limit the number of genes to < 9000. Treated samples cluster together with uninfected samples. **e** DESeq2 analysis of infected, MK-4482-treated vs vehicle-treated samples identified significant (adjusted *p* value < 0.1 and |log2 of the fold change|> 1) down regulation of 66 genes after MK-4482 treatment compared to the infected vehicle-treated controls. Reactome analysis of the 66 genes is shown as a bar plot with −log10(fdr) as *x*-axis. **f**, **g** Lungs harvested from the three groups of hamsters were stained with hematoxylin and eosin (H&E). Representative images (500 mg/kg dose) are shown in **f**. Scale bar = 250 µm. Violin plots in **g** display the abundance of cellularity and infiltrates in the lungs of the three groups, as determined by ImageJ. Medians are indicated with a bar. A nonparametric, two-sided Mann–Whitney test was used to determine significance. Source data are provided as a Source Data file.

Due to the demonstrated in vitro potency in the ALI-HBEC primary cell model and adequate exposures of nelfinavir and MK-4482/N-hydroxycytidine (a time over Calu-3 SARS-CoV-2 EC_50_ of ~3 h for a single 500 mg/kg PO dose of nelfinavir, and time over HeLa-ACE2 and Calu-3 EC_50_ ≥ 7 h for a single 500 mg/kg PO dose of MK-4482 (Supplementary Fig. 3a, b and Supplementary Table 1)), we investigated the efficacy of nelfinavir and MK-4482 in a Golden Syrian hamster animal model of SARS-CoV-2 infection (Fig. 4a). Nelfinavir was delivered PO at 500 mg/kg BID (twice daily) and MK-4482 was delivered PO at 500 mg/kg, 150 mg/kg, and 50 mg/kg BID, to evaluate dose-dependent protection. A matched vehicle-only suspension was used as a control. Four hours after first treatment, animals were challenged with 1 × 10^6^ PFU of SARS-CoV-2 (USA-WA1/2020) by intranasal administration. The animals were weighed daily as a measure of disease progression and lung tissue was isolated on day 5 of infection to determine viral titers, lung histology, and gene expression profiles.

Nelfinavir failed to protect animals from weight loss and viral replication (Supplementary Fig. 3c, d), potentially due to inadequate plasma exposure in hamsters (nelfinavir failed to reach exposures above HeLa-ACE2 SARS-CoV-2 EC_50_ (Supplementary Fig. 3b)). However, MK-4482 protected animals from severe weight loss at 500 mg/kg, with this treatment group averaging 97% of their starting weight at day 5 of infection (Fig. 4b). The 150 mg/kg and 50 mg/kg groups showed partial protection through weight loss, averaging 89% and 90% of their starting weight, respectively, compared to the vehicle control 85% at day 5 of infection (Fig. 4b).

To analyze correlates to weight loss, the relative virus titers were determined from day 5 lung samples using a crystal violet-based plaque assay. The 500 mg/kg and 150 mg/kg doses had undetectable live viral titers in the lungs, showing full protection from virus replication (Fig. 4c). The 50 mg/kg group averaged 4.5 × 10^3^ PFU/lung, showing moderately good efficacy (99% viral reduction) compared to the vehicle control group which averaged 4.5 × 10^5^ PFU/lung (Fig. 4c).

Protection from weight loss and viremia in the 500 mg/kg treatment arm was associated with a near-complete protection from host immune response, as determined by RNA-Seq analysis on hamster lungs followed by unsupervised hierarchical clustering; the MK-4482-treated (500 mg/kg) samples clustered together with uninfected samples (Fig. 4d). A DESeq2 analysis confirmed that infected vehicle-treated lungs induce the expression of 66 genes associated with pathways reported to be upregulated in COVID-19, including interferon signaling and interferon stimulated genes^28,29^ (Fig. 4e). Finally, histological examination confirmed that the lungs from MK-4482-treated hamsters were protected and more closely resembled those tissues from uninfected animals. In stark contrast, examination of lung tissue in the vehicle-treated control group revealed obliteration of alveolar spaces and overwhelming immune cell infiltration (Fig. 4f, g).

## Discussion

In summary, by exhaustive screening of the high-value repurposing ReFRAME library, we identified the oral nucleoside analog MK-4482 to have the highest potential for repurposing and rapid development as a COVID-19 therapy. This compound with broad antiviral activity is currently in a phase II/III clinical trial led by Ridgeback Biotherapeutics and Merck to evaluate its safety and efficacy and recently has shown efficacy in a SARS-CoV-2 ferret^26^ and humanized mouse^27^ models of infection. Other identified hits may have value as combination therapies or tool compounds and deserve additional investigation.

In total, we identified 90 unique known drugs or preclinical molecules with activity against SARS-CoV-2 in human cells, 19 of which were tested and showed an additive interaction in combination with the FDA-approved antiviral compound remdesivir (Veklury®). We also identified a synergistic interaction between remdesivir and both 10-deazaaminopterin and riboprine. Our data support the advancement of the identified compounds for further profiling in in vivo models to assess their utility (alone or in combination with remdesivir) in combating the COVID-19 pandemic.

The recent emergency use authorization of safe and effective SARS-CoV-2 vaccines has the potential to dramatically limit SARS-CoV-2 infections. However, shortcomings in vaccine supply, public compliance, unknown duration of immunity, and potential for mutation of vaccine targets all suggest the need for further development of easily accessible drug interventions (e.g. once or twice daily self-administered, oral treatment), for treatment of symptomatic COVID-19 patients and to limit viral transmission. Repurposing of existing therapeutics presents the shortest path for drug development. Yet, the ReFRAME library, which is a fair representation of the world’s pharmacopeia, yielded limited high-value candidates for drug repurposing. Among those identified candidates, some have already been rapidly advanced into clinical trials, such as hydroxychloroquine, and did not produce significant clinical benefit. This limitation in high-value drug repurposing candidates for SARS-CoV-2 infection reflects the paucity of approved and advanced antivirals/anti-infectives, and further highlights the need for focused drug discovery development. Review of the ReFRAME library composition underscores this need because only 14% of its compounds are classified as anti-infectives. Investments in this area will not only develop more efficacious treatments for COVID-19 but will provide advanced chemical matter for existing and emerging health crises, accelerating the global response. Our validated in vitro and in vivo workflow and assays have the potential to aid in development and evaluation of such chemical entities for treatment of this and future pandemics.

## Methods

### Virus generation

Vero E6 cells (ATCC CRL-1586) were plated in a T225 flask with complete DMEM (Corning 15-013-CV) containing 10% FBS, 1×PenStrep (Corning 20-002-CL), 2 mM l-Glutamine (Corning 25-005-CL) overnight at 37 °C 5% CO_2_. The media in the flask was removed and 2 mL of SARS-CoV-2 strain USA-WA1/2020 (BEI Resources NR-52281) in complete DMEM was added to the flask at an MOI of 0.5 and allowed to incubate for 30 min at 34 °C 5% CO_2_. After incubation, 30 mL of complete DMEM was added to the flask. The flask was then placed in a 34 °C incubator at 5% CO_2_ for 5 days. On day 5 post-infection the supernatant was harvested and centrifuged at 1000×*g* for 5 min. The supernatant was filtered through a 0.22 µm filter and stored at −80 °C.

### The ReFRAME library: compound management, drug annotation and screen data access

The ReFRAME library collection consists of nearly 12,000 high-purity compounds (> 95%) dissolved in high-quality dimethyl sulfoxide (DMSO) and has been previously described^10^. Compound quality control was performed by liquid chromatography-mass spectrometry and/or ^1^H-NMR when required. The library was prepared at two concentrations, 2 and 10 mM, to support low-concentration (2–10 µM) and high-concentration (10–50 µM) screening formats. Echo-qualified 384-well low dead volume plus microplates (LP-0200-BC; Labcyte Inc.) were used as the library source plates to support acoustic transfer with an Echo 555 Liquid Handler (Labcyte Inc.). Compounds not soluble in DMSO were plated in water (129 compounds); compounds lacking long-term solubility in DMSO were suspended just before dispensing to avoid precipitation (71 compounds). Additional details available at https://reframedb.org/about.

Associated compound annotation are supported by three widely used commercial drug competitive intelligence databases: Clarivate Integrity, GVK Excelra GoStar, and Citeline Pharmaprojects. As available, annotation data may include status of clinical development and highest stage of development achieved, mechanism of action, drug indication(s), and route of administration.

In accordance with ReFRAME data policies, open access to these data is assured and have been expedited for immediate disclosure at https://reframedb.org/.

### HeLa-ACE2 stable cell line

HeLa-ACE2 cells were generated through transduction of human ACE2 lentivirus. The lentivirus was created by co-transfection of HEK293T cells with pBOB-hACE2 construct and lentiviral packaging plasmids pMDL, pREV, and pVSV-G (Addgene) using Lipofectamine 2000 (Thermo Fisher Scientific, 11668019). Supernatant was collected 48 h after transfection then used to transduce pre-seeded HeLa cells. In all, 12 h after transduction stable cell lines were collected, scaled up, and stored. Cells were maintained in DMEM (Gibco, 11965-092) with 10% FBS (Gibco, 10438026) and 1× sodium pyruvate (Gibco, 11360070) at 37 °C 5% CO_2_.

### SARS-CoV-2/HeLa-ACE2 high-content screening assay

Compounds were acoustically transferred into 384-well µclear-bottom plates (Greiner, Part. No. 781090-2B). HeLa-ACE2 cells were seeded in 13 µL DMEM with 2% FBS at a density of 1.0 × 10^3^ cells per well. Plated cells were transported to the BSL3 facility where 13 µL of SARS-CoV-2 diluted in assay media was added per well at an assay multiplicity of infection (MOI) = 2.2 for primary screening, adjusted to 0.65 for powder reconfirmation. Plates were incubated for 24 h at 34 °C 5% CO_2_, and then fixed with final concentration of 4% formaldehyde for 1 h at 34 °C 5% CO_2._ Plates were washed with 1xPBS 0.05% Tween 20 in between fixation and subsequent primary and secondary antibody staining. Human polyclonal plasma (a unique and limited reagent that can be supplied in 500 µL quantities) diluted 1:500 in Perm/Wash buffer (BD Biosciences 554723) was added to the plate and incubated at RT for 2 h. In total, 8 µg/mL (a 1:250 dilution) of goat anti-human H + L conjugated Alexa 488 (Thermo Fisher Scientific A11013) together with 3 µM of antifade-46-diamidino-2-phenylindole (DAPI; Thermo Fisher Scientific D1306) in SuperBlock T20 (PBS) buffer (Thermo Fisher Scientific 37515) was added to the plate and incubated at RT for 1 h in the dark. Plates were imaged using the ImageXpress Micro Confocal High-Content Imaging System (Molecular Devices) with a ×10 objective, with 4 fields imaged per well. Images were analyzed using the Multi-Wavelength Cell Scoring Application Module (MetaXpress), with DAPI staining identifying the host-cell nuclei (the total number of cells in the images) and the SARS-CoV-2 immunofluorescence signal leading to identification of infected cells.

### Time of addition assay

HeLa-ACE2 cells were infected with SARS-CoV-2 in suspension in assay medium (DMEM with 2% FBS) at an MOI of 1.5 for 1 h at 34 °C 5% CO_2_, then extensively washed with PBS and plated in assay-ready 384-well plates pre-spotted with compounds as for the standard HeLa-ACE2 infection assay. For the time course experiment, cells were fixed with a final concentration of 4% formaldehyde at 4, 5, 6, 7, 8, 10, 11, 12, and 24 hpi and stained and imaged as for the standard infection assay to determine optimal timepoint for TOA assay. TOA assay was performed in the same manner, with cells fixed at 10 hpi.

### Calu-3 high-content screening assay

The assay is carried out as outlined for the HeLa-ACE2 assay, with the following exceptions. Calu-3 cells (ATCC HTB-55), a kind gift from Dr. Catherine Chen at NCATS/NIH and Dr. Juan Carlos de la Torre at Scripps Research, were seeded at a density of 5000 cells per 20 µL per well in assay media (MEM with 2% FBS) and SARS-CoV-2 diluted in assay media was added at an MOI between 0.75 and 1 to achieve ~30–60% infected cells. Plates were incubated for 48 h at 34 °C 5% CO_2_, and then fixed with a final concentration of 4% formaldehyde. Fixed cells were stained and imaged as in the HeLa-ACE2 assay.

### Uninfected host-cell cytotoxicity counter screens

For HeLa-ACE2 cells, compounds were acoustically transferred into 1,536-well µclear plates (Greiner Part. No. 789091). HeLa-ACE2 cells were maintained as described for the infection assay and seeded in the assay-ready plates at 400 cells/well in DMEM with 2% FBS and plates were incubated for 24 h at 37 °C 5% CO_2_. To assess cell viability, the Image-iT DEAD green reagent (Thermo Fisher) was used according to manufacturer instructions. Cells were fixed with 4% paraformaldehyde, and counterstained with DAPI. Fixed cells were imaged using the ImageXpress Micro Confocal High-Content Imaging System (Molecular Devices) with a ×10 objective, and total live cells per well quantified in the acquired images using the Live Dead Application Module (MetaXpress).

For Calu-3 cells, compounds were acoustically transferred into 1,536-well plates (Corning No. 9006BC) before seeding Calu-3 cells in assay media (MEM with 2% FBS) at a density of 600 cells per 5 µL per well. Plates were incubated for 48 h at 37 °C 5% CO_2_. To assess cell viability, 2 µL of 50% Cell-Titer Glo (Promega No G7573) diluted in water was added to the cells and luminescence measured on an EnVision Plate Reader (Perkin Elmer).

HepG2 (ATCC HB-8065) and HEK293T (ATCC CRL-3216) mammalian cell lines were maintained in Dulbecco’s Modified Eagle Medium (DMEM, Gibco) with 10% heat-inactivated HyClone FBS (GE Healthcare Life Sciences), 100 IU penicillin, and 100 μg/mL streptomycin (Gibco) at 37 °C with 5% CO_2_ in a humidified tissue culture incubator. To assay mammalian toxicity of hit compounds, 750 HepG2 and 375 HEK293T cells/well were seeded, respectively, in assay media (DMEM, 2% FBS, 100 IU penicillin, and 100 μg/mL streptomycin) in 1536-well, white, tissue culture-treated, solid bottom plates (Corning, 9006BC) that contained acoustically transferred compounds in a three-fold serial dilution starting at 40 µM. After a 72-h incubation, CellTiter-Glo Luminescent Cell Viability Assay (Promega No G7573) was used to quantify cell viability as for Calu-3 cells.

### SARS-CoV-2 primary ALI HBEC model

Normal primary human bronchial epithelial cells (HBECs) (Lonza) were cultured in Millicell-96 cell culture insert plates with 1 µm PET filters (Sigma) at an air–liquid interface for at least 4 weeks using PneumaCult™-ALI Medium (Stemcell Technologies). Briefly, the HBECs were first expanded in cell culture flasks before seeding 10,000 cells per well submerged in PneumaCult™-Ex Plus Medium. After 1 week, the cells were switched into PneumaCult™-ALI Medium and medium was removed from the apical surface. The air–liquid interface was maintained, and the medium exchanged every 2−3 days for at least 4 weeks to allow for differentiation of the cells. Prior to infection, the apical surface was rinsed once with DPBS and compounds were added to the basolateral chamber. In all, 20,000 PFU SARS-CoV-2 strain USA-WA1/2020 were added to the apical surface in 50 µL PBS and allowed to incubate for 2 h. The inoculum was then removed, and the cells rinsed once with DPBS. The medium was exchanged, and fresh compound added at 24 and 48 h post-infection. Apical washes were collected at 72 h post-infection by adding 100 µL DPBS to the apical surface for 15 minutes. RNA was isolated from the apical washes using the PureLink™ Pro 96 Viral RNA/DNA Purification Kit (Thermo Fisher) and analyzed for viral RNA levels by RT-qPCR using the SuperScript™ III Platinum™ One-Step qRT-PCR Kit (Thermo Fisher) and the 2019-nCoV N1 CDC Primers and Probe set (Integrated DNA Technologies) (primer details in Supplementary Table 2). A standard curve was generated by isolating RNA from serial dilutions of the stock virus and used to determine the PFU equivalents/mL for each sample. The viral load reductions were then determined for each experimental compound treatment compared to the neutral DMSO control and plotted in log scale. Cytotoxicity was assessed by measuring LDH activity in the basolateral media using a Cytotoxicity Detection kit (LDH) (Sigma) following the manufacturer’s instructions. Averages were taken for the experimental samples and presented as a percentage of the positive control puromycin. Technical triplicates were run for both antiviral and cytotoxicity readouts.

### Golden Syrian Hamster SARS-CoV-2 efficacy model

Eight-week-old Golden Syrian hamsters (Charles River) (five per group) were dosed *per os* (PO) as indicated. Four hours post first dose, hamsters were infected through intranasal installation of 10^6^ total PFU per animal in 100 µL of DMEM. Hamsters were dosed with compound bidaily (BID) and weighed for the duration of the study. At day 5 post-infection, the hamsters were sacrificed, and lung tissue was isolated for analysis. The research protocol was approved and performed in accordance with Scripps Research IACUC Protocol #20-0003.

### Lung viral titer determination

SARS-CoV2 titers were measured by homogenizing organs in DMEM 2% FCS using 100 µm cell strainers (Myriad 2825-8367). Homogenized organs were titrated 1:10 over six steps and layered over Vero cells. After 1 h of incubation at 37 °C, a 1% methylcellulose in DMEM overlay was added, and the cells were incubated for 3 days at 37 °C. Cells were fixed with 4% PFA and plaques were counted by crystal violet staining.

### Pharmacokinetic studies

Pharmacokinetic studies were conducted at Scripps Research Institute’s Animal Models Core in accordance with IACUC guidelines (IACUC Protocol #09-0004-5). Eight-week-old male Syrian Hamsters (Charles River; three per group) were dosed PO as indicated for each compound and formulation. Plasma concentration of each test article was monitored up to 48 h. Nelfinavir was formulated in 10% DMSO/90% corn oil and MK-4482 was formulated in 10% PEG400/2.5% Cremaphor RH40 for both pharmacokinetic and efficacy studies.

### Hamster lung RNA analysis

Hamster lung from uninfected (U, *n* = 2), vehicle treated (V, *n* = 4), and MK-4482 treated (T, *n* = 4) samples were analyzed using RNA-Seq platform. Mean absolute deviation (MAD) is computed for all genes using python package scipy.stats.median_absolute_deviation. StepMiner algorithm^30^ was applied to select the high MAD values which filter 22,284 genes down to 14,939. StepMiner algorithm was applied again to filter 14,939 down to 8617 genes. Hierarchical agglomerative clustering analysis was performed on these 8617 genes with python seaborn clustermap library function. Differential expression analysis is performed using DESeq2^31^ (MK-4482 treated vs the vehicle-treated samples) and adjusted *p* value < 0.1 and |log2 of the fold change| > 1 is applied to identify up/down regulated genes. Reactome pathway analysis^32^ of differentially expressed genes was performed to identify the high-level the biological processes enriched in the gene set. A bar plot with −log10(fdr) as *x*-axis is used to demonstrate the significance of the enriched biological processes.

### RNA-Seq

RNA sequencing libraries were generated using the Illumina TruSeq Stranded Total RNA Library Prep Gold with TruSeq Unique Dual Indexes (Illumina, San Diego, CA) exactly as described before^29^. Samples were processed following manufacturer’s instructions, except modifying RNA shear time to 5 min. Resulting libraries were multiplexed and sequenced with 100 basepair (bp) Paired End (PE100) to a depth of approximately 25–40 million reads per sample on an Illumina NovaSeq 6000 by the Institute of Genomic Medicine (IGM) at the University of California San Diego. Samples were demuxltiplexed using bcl2fastq v2.20 Conversion Software (Illumina, San Diego, CA). RNA-Seq data was processed using kallisto (version 0.45.0), *Mesocricetus auratus* genome (MesAur1.0). Gene-level TPM values and gene annotations were computed using tximport and biomaRt R package. A custom python script was used to organize the data and log reduced using log2(TPM) if TPM > 1 and TPM - 1 if TPM ≤  1. For the hamster study kallisto index was prepared on Mesocricetus_auratus.MesAur1.0.ncrna.fa.gz + Mesocricetus_auratus MesAur1.0 cdna.all.fa.gz. The raw data and processed data are deposited in Gene Expression Omnibus (pending GSEID from NCBI GEO).

### Hamster lung histopathology/infiltrate quantification

ImageJ (V1.53) software is used to quantify H&E-stained slide images (at ×20 magnification). Images are first converted to 8-Bit (Image > Type > 8-Bit), their threshold is adjusted (Image > Adjust > Threshold), a threshold value between 70–80% is chosen to ensure only dark stained nuclei are detected. Following thresholding the image is converted to a mask (Process > Binary > Convert to Mask) and analyzed (Analyze > Analyze Particles) with default settings, adding display results and show outline and the output was exported into GraphPad Prism (V9.0.0) where the nonparametric, two-sided Mann–Whitney statistical test was used to calculate significance.

### Data analysis

High-content image analysis was carried out with MetaXpress (version 6.5.4.532). Primary in vitro screen and the host-cell cytotoxicity counter screen data were uploaded to Genedata Screener, Version 16.0.3-Standard. HeLa-ACE2 data were normalized to neutral (DMSO) minus inhibitor controls (2.4 µM remdesivir for antiviral effect in HeLa-ACE2 cells and 9.6 µM puromycin for infected host-cell toxicity). Calu-3 infection assay data were normalized to neutral (DMSO) minus inhibitor control (10 µM remdesivir), and for the Calu-3 cell count readout the total cells were normalized to the stimulator (10 µM remdesivir) minus neutral control (DMSO). For the uninfected host-cell cytotoxicity counter screens, 40 µM puromycin (Sigma) was used as the positive (inhibitory) control in HeLa-ACE2, HepG2, and HEK293T cells, and 30 µM puromycin (Sigma) was used as the positive (inhibitory) control for Calu-3 cells. For dose-response experiments compounds were tested in technical triplicates on different assay plates and dose curves were fitted with the four parameter Hill Equation. Technical replicate data were analyzed using median condensing. Geometric means and geometric standard deviations are reported for compound activities (EC_50_s and CC_50_s) obtained in multiple independent biological experiments. The *synergyfinder* package^13^ in R (version 3.6.3) was used for synergy analysis. Geometric means were calculated by computing the logarithm (base 10) of all values, calculating the mean of these logarithms, and taking the antilog of that mean. Geometric standard deviations were computed by taking the standard deviation of the log-transformed individual values and taking the antilog of that standard deviation. The geometric standard deviation is a unitless ratio and reported as ×÷ instead of ±. That is, for a reported 0.123 µM ×÷ 1.276, the standard deviation range is from 0.096 µM to 0.157 µM (i.e. 0.123 µM ÷ 1.276 to 0.123 µM × 1.276).

### Reporting summary

Further information on research design is available in the Nature Research Reporting Summary linked to this article.

## Supplementary information

Supplementary Information

Description of Additional Supplementary Files

Supplementary Data 1

Supplementary Data 2

Reporting Summary

## Data Availability

All data are available in the main text or the supplementary materials. Results from the screens of the ReFRAME library have been deposited to the reframedb.org data portal (HeLa-ACE2 assay numbers: A00466, A00485, A00488, and Calu-3 assay numbers: A00527, A00529, A00541). RNA-Seq raw and processed data that support the findings of this study have been deposited in Gene Expression Omnibus with the accession code GSE168095. Source data are provided with this paper.

## Source data

Source Data

## Code availability

Custom Python (V 3.6.9) scripts used in the manuscript can be found in GitHub: Source: https://github.com/sahoo00/BoNE (cd BoNE); https://github.com/sahoo00/Hegemon (javac Hegemon/tools/*.java, cd namir, bash scr).
