# Supplementary material for: Drug repurposing screens identify chemical entities for the development of COVID-19 interventions
Source: Nat Commun. 2021 Jun 3;12:3309. doi: 10.1038/s41467-021-23328-0 (PMC8175350; doi:10.1038/s41467-021-23328-0)
Supplement: Supplementary file 1 — Supplementary Information [file 41467_2021_23328_MOESM1_ESM.pdf]

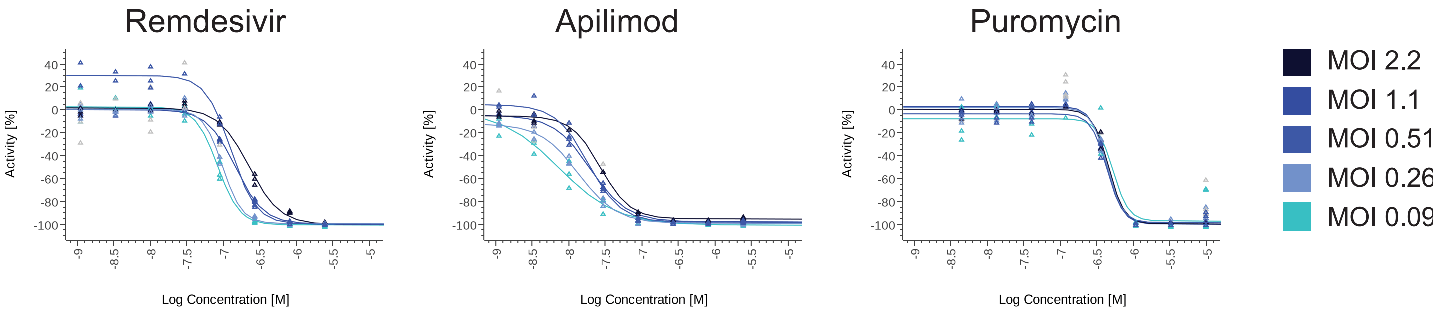

Remdesivir	Apilimod	Puromycin
MOI 2.2: 223 nM	MOI 2.2: 27 nM	MOI 2.2: 455 nM
MOI 1.1: 145 nM	MOI 1.1: 19.6 nM	MOI 1.1: 431 nM
MOI 0.51: 126 nM	MOI 0.51: 18.5 nM	MOI 0.51: 430 nM
MOI 0.26: 97 nM	MOI 0.26: 13.6 nM	MOI 0.26: 433 nM
MOI 0.09: 83 nM	MOI 0.09: 7.3 nM	MOI 0.09: 520 nM

Supplementary Fig. 1.

Effects of MOI on control compound EC_{50}s. Activity of remdesivir, apilimod, and puromycin controls in the SARS-CoV-2/HeLa-ACE2 assay was assessed with MOIs ranging from 0.09 to 2.2. EC_{50} of each compound at the indicated MOI is shown. Source data are provided as a Source Data file.

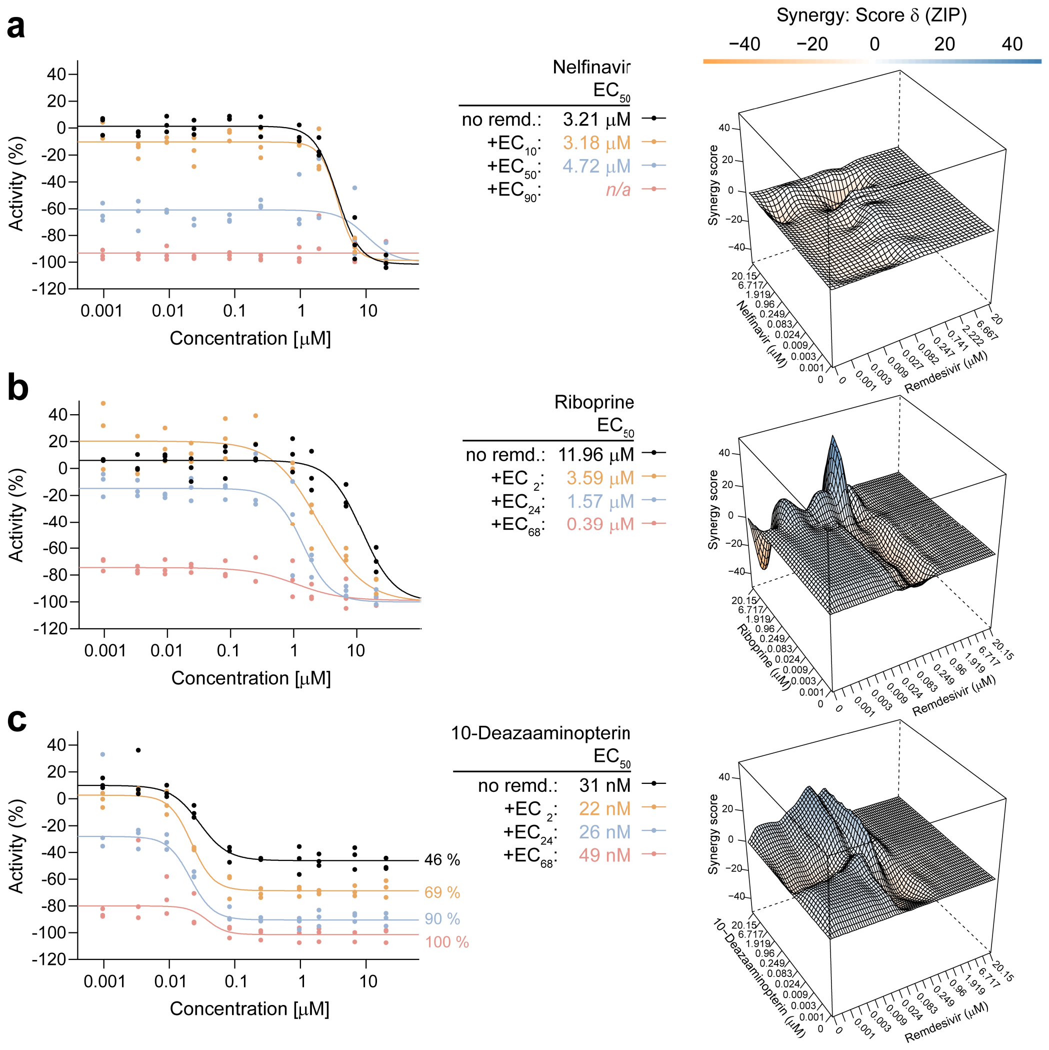

Supplementary Fig. 2.

Anti-SARS-CoV-2 activity of drugs in combination with remdesivir showing additive and synergistic interactions. Dose response curves for nelfinavir (**a**), riboprine (**b**) and 10-deazaaminopterin (**c**) without or with increasing concentrations of remdesivir as well as the output of the synergy analysis, a 3-dimensional drug interaction landscape plotting synergy scores across all compound concentrations tested (median scores of 3 technical replicates) are shown. Additive effect: -10< δ <10; synergistic effect: δ >10; antagonistic effect: δ <10. SARS-CoV-2 EC_{50} of each compound in combination with varying concentrations of remdesivir is also shown. Effective concentrations for remdesivir were calculated based on activity of remdesivir in each experiment. Source data are provided as a Source Data file.

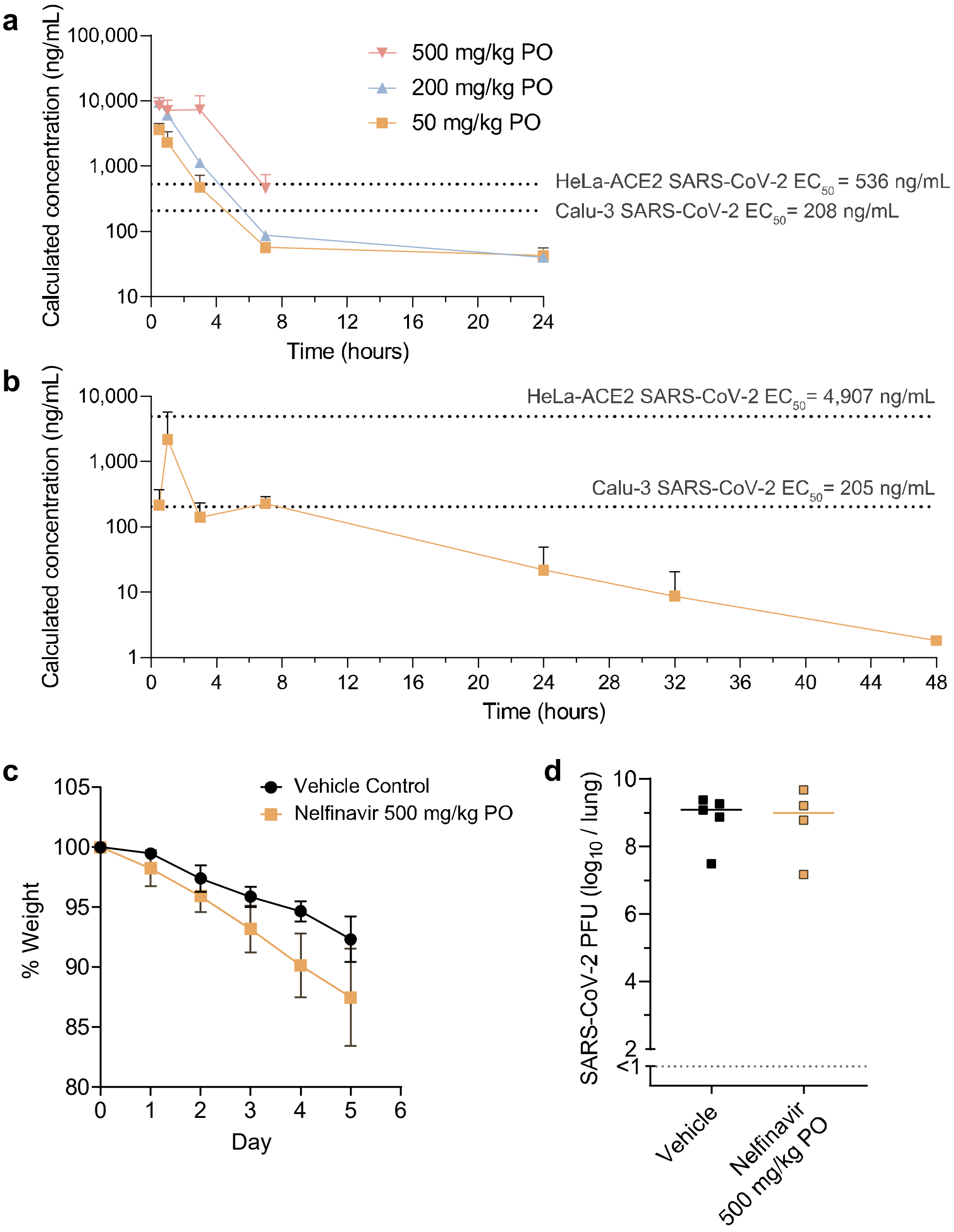

Supplementary Fig. 3.

Hamster PK and nelfinavir *in vivo* results. PK of MK-4482 (**a**) and nelfinavir (**b**) was evaluated after a single oral dose of each compound as indicated. HeLa-ACE2 and Calu-3 SARS-CoV-2 EC_{50}s are shown for comparison. For MK-4482 prodrug experiment levels of N-hydroxycytidine metabolized parent nucleoside are shown. For **a**, **b**, means ± s.d. are shown for n=3 animals per group examined over one independent experiment. Effect of a 500 mg/kg PO nelfinavir treatment on hamster weights, means ± s.d. are shown. (**c**) and viral titers in lungs, median and individual values (**d**) are shown. For **c**, **d**, n=5 animals per vehicle and n=4 animals per nelfinavir group were examined over one independent experiment. Source data are provided as a Source Data file.

Supplementary Table 1.
Summary of pharmacokinetics in Syrian Hamsters of compounds administered PO (*per os*).

Dose (mg/kg)	Compound	Formulation	$t_{1/2}$ (h)	C_{max} (ng/mL)	T_{max} (h)	AUC_{0-24} (hr*ng/mL)	AUC_{LAST} (hr*ng/mL)	AUC_{INF} (hr*ng/mL)	Vd (L/kg)	Cl (mL/min/kg)	MRT (h)
500	Nelfinavir mesylate	10%DMSO/90% Corn Oil	4.02	2,252.67	2.83	3,762.49	3,874.46	5,479.02	706.35	2,542.16	5.82
50	MK-4482 (N-hydroxycytidine measured)	10%PEG400/2.5% Cremaphor RH40		3,823.33	0.67	6,260.00	8,423.33				11.76
200	MK-4482 (N-hydroxycytidine measured)	10%PEG400/2.5% Cremaphor RH40		116,816.67	32.17	14,700.00	927,933.33				35.03
500	MK-4482 (N-hydroxycytidine measured)	10%PEG400/2.5% Cremaphor RH40	2.41	8,706.67	1.33	30,866.67	29,600.00	20,800.00	83.60	401.00	2.45

Supplementary Table 2.
Information and sequences of primers and probe for RT-qPCR.

Primer/Probe	Supplier, Catalog #	Sequence
nCOV_N1 Forward Primer Aliquot, 100 nmol	IDT, cat #10006830	GACCCCAAAATCAGCGAAAT
nCOV_N1 Reverse Primer Aliquot, 100 nmol	IDT, cat #10006831	TCTGGTTACTGCCAGTTGAATCTG
nCOV_N1 Probe Aliquot, 50 nmol: 5' FAM / 3' Black Hole Quencher® (BHQ) probes	IDT, cat # 10006832	FAM-ACCCCGCATTACGTTTGGTGGACC-3IABkFQ
