# Supplementary material for: Drug repurposing screens identify chemical entities for the development of COVID-19 interventions
Source: Nat Commun. 2021 Jun 3;12:3309. doi: 10.1038/s41467-021-23328-0 (PMC8175350; doi:10.1038/s41467-021-23328-0)
Supplement: Supplementary file 2 — Description of Additional Supplementary Files [file 41467_2021_23328_MOESM2_ESM.pdf]

Description of Additional Supplementary Files

File Name: Supplementary Data 1
Description: Anti-SARS-CoV-2 activities of potent and selective hits in the pilot and ReFRAME HeLa-ACE2 in vitro screen.

File Name: Supplementary Data 2
Description: Anti-SARS-CoV-2 activities of potent and selective hits in the ReFRAME Calu-3 in vitro screen.
