# Supplementary material for: Drug repurposing screens identify chemical entities for the development of COVID-19 interventions
Source: Nat Commun. 2021 Jun 3;12:3309. doi: 10.1038/s41467-021-23328-0 (PMC8175350; doi:10.1038/s41467-021-23328-0)
Supplement: Supplementary file 5 — Reporting Summary [file 41467_2021_23328_MOESM5_ESM.pdf]

Corresponding author(s): Malina Bakowski, Thomas Rogers

Last updated by author(s): Apr 13, 2021

Reporting Summary

Nature Research wishes to improve the reproducibility of the work that we publish. This form provides structure for consistency and transparency in reporting. For further information on Nature Research policies, see our Editorial Policies and the Editorial Policy Checklist.

Statistics

For all statistical analyses, confirm that the following items are present in the figure legend, table legend, main text, or Methods section.

n/a	Confirmed	
☐	☒	The exact sample size (*n*) for each experimental group/condition, given as a discrete number and unit of measurement
☐	☒	A statement on whether measurements were taken from distinct samples or whether the same sample was measured repeatedly
☐	☒	The statistical test(s) used AND whether they are one- or two-sided<br>*Only common tests should be described solely by name; describe more complex techniques in the Methods section.*
☒	☐	A description of all covariates tested
☐	☒	A description of any assumptions or corrections, such as tests of normality and adjustment for multiple comparisons
☐	☒	A full description of the statistical parameters including central tendency (e.g. means) or other basic estimates (e.g. regression coefficient) AND variation (e.g. standard deviation) or associated estimates of uncertainty (e.g. confidence intervals)
☐	☒	For null hypothesis testing, the test statistic (e.g. F, t, r) with confidence intervals, effect sizes, degrees of freedom and P value noted<br>*Give P values as exact values whenever suitable.*
☒	☐	For Bayesian analysis, information on the choice of priors and Markov chain Monte Carlo settings
☒	☐	For hierarchical and complex designs, identification of the appropriate level for tests and full reporting of outcomes
☒	☐	Estimates of effect sizes (e.g. Cohen's d, Pearson's r), indicating how they were calculated

Our web collection on statistics for biologists contains articles on many of the points above.

Software and code

Policy information about availability of computer code

Data collection	MetaXpress (version 6.5.4.532) software was used to analyze images acquired in high-content screening. RNA was sequenced on an Illumina NovaSeq 6000 and samples were demuxltiplexed using bcl2fastq v2.20 Conversion Software (Illumina).
Data analysis	Genedata Screener, Version 16.0.3-Standard and synergyfinder package in R (version 3.6.3) were used to analyze the screening data. ImageJ (V1.53) software is used to quantify H&E stained slide images. GraphPad Prism (V3.6.9) was used to graph and analyze select data. RNASeq data was processed using kallisto (version 0.45.0). Gene-level TPM values and gene annotations were computed using tximport and biomaRt R package. Mean absolute deviation (MAD) for RNAseq analysis was computed for all genes using python package scipy.stats.median_absolute_deviation and the StepMiner algorithm was applied to select the high MAD values. Hierarchical agglomerative clustering analysis was performed with python seaborn clustermap library function. Differential expression analysis was performed using DESeq. Reactome pathway analysis was performed to identify the enriched high-level biological processes.<br>Custom Python (V 3.6.9) scripts used in the manuscript can be found in GitHub:<br>Source: https://github.com/sahoo00/BoNE (cd BoNE)<br>Source: https://github.com/sahoo00/Hegemon (javac Hegemon/tools/*.java, cd namir, bash scr)

For manuscripts utilizing custom algorithms or software that are central to the research but not yet described in published literature, software must be made available to editors and reviewers. We strongly encourage code deposition in a community repository (e.g. GitHub). See the Nature Research guidelines for submitting code & software for further information.

Data

Policy information about availability of data

All manuscripts must include a data availability statement. This statement should provide the following information, where applicable:

- Accession codes, unique identifiers, or web links for publicly available datasets
- A list of figures that have associated raw data
- A description of any restrictions on data availability

All data are available in the main text or the supplementary materials. Results from the screens of the ReFRAME library have been deposited to the reframedb.org data portal (HeLa-ACE2 assay numbers: A00466, A00485, A00488 and Calu-3 assay numbers: A00527, A00529, A00541). RNAseq raw and processed data that support the findings of this study have been deposited in Gene Expression Omnibus with the accession code GSE168095. Source data are provided with this paper.

Field-specific reporting

Please select the one below that is the best fit for your research. If you are not sure, read the appropriate sections before making your selection.

☒ Life sciences ☐ Behavioural & social sciences ☐ Ecological, evolutionary & environmental sciences

For a reference copy of the document with all sections, see nature.com/documents/nr-reporting-summary-flat.pdf

Life sciences study design

All studies must disclose on these points even when the disclosure is negative.

Sample size	We routinely used technical triplicates when reconfirming activity of compounds in dose response. That sample size was chosen to enable arbitration between divergent data for specific compound concentrations but without limiting assay throughput. For in vivo experiments the sample size used (n=5) was found to be sufficient to assess statistical significance of viral titers.
Data exclusions	Outlier data in technical replicates was excluded from dose response analysis by standard settings of the GeneData Screener curve fitting algorithm. The exclusion criteria were predetermined.
Replication	We tracked control compound activity and the assay quality statistics to ensure screening data was reproducible across assay runs. Activity of controls was reproducible. Not all hits identified in the single point primary screen reconfirmed as active compounds in dose response reconfirmation, but % reconfirmation was within acceptable levels: ~40% reconfirmation is usually expected in a high-throughput screen and the reconfirmation rate for this ReFRAME primary screen was >70%, indicating above average quality of both the assay and the ReFRAME library. For each experiment, the number of replicates performed independently is indicated in figure legends and Supplementary Data 1 and Supplementary Data 2. For powder reconfirmation most assays/compounds were tested at least three independent times. For in vivo assays, experiments were performed one independent time.
Randomization	Due to technical challenges involved we did not actively randomize compound distribution in plates and plate control layout was the same for each type of experiment (however test article distribution was randomly assigned). No clear patterns with respect to location of compounds in plates were observed. For in vivo experiments animals were assigned to study groups at random.
Blinding	Data collection (high-content imaging) was carried out in an automated high-throughput fashion and independent of investigator input. Compounds were screened in a high-throughput manner and due to compound registration it was impossible to analyze the results in a completely blinded fashion. In vivo experiment data collection was blinded and investigators were blinded to group allocation during data analysis.

Reporting for specific materials, systems and methods

We require information from authors about some types of materials, experimental systems and methods used in many studies. Here, indicate whether each material, system or method listed is relevant to your study. If you are not sure if a list item applies to your research, read the appropriate section before selecting a response.

Materials & experimental systems

n/a	Involved in the study	
☐	☒	Antibodies
☐	☒	Eukaryotic cell lines
☒	☐	Palaeontology and archaeology
☐	☒	Animals and other organisms
☒	☐	Human research participants
☒	☐	Clinical data
☒	☐	Dual use research of concern

Methods

n/a	Involved in the study	
☒	☐	ChIP-seq
☒	☐	Flow cytometry
☒	☐	MRI-based neuroimaging

Antibodies

Antibodies used	Human polyclonal sera from COVID-19 patients; goat anti-human H+L conjugated Alexa 488 (Thermo Fisher Scientific, A11013)
Validation	Human polyclonal sera from COVID-19 patients was screened for uninfected cell staining and other off target staining. It was confirmed that the sera specifically stained SARS-CoV-2 infected cells.<br>According to the manufacturer, to minimize cross-reactivity, the goat anti-human IgG (H+L) whole secondary antibodies have been affinity purified and cross-adsorbed against mouse, rabbit, and bovine serum prior to conjugation. Cross-adsorption or pre-adsorption is a purification step to increase specificity of the antibody resulting in higher sensitivity and less background staining. The secondary antibody solution is passed through a column matrix containing immobilized serum proteins from potentially cross-reactive species. Only the nonspecific-binding secondary antibodies are captured in the column, and the highly specific secondaries flow through. The benefits of this extra step are apparent in multiplexing/multicolor-staining experiments (e.g., flow cytometry) where there is potential cross-reactivity with other primary antibodies or in tissue/cell fluorescent staining experiments where there are may be the presence of endogenous immunoglobulins.

Eukaryotic cell lines

Policy information about cell lines

Cell line source(s)	Vero E6 (ATCC CRL-1586); HeLa-ACE2 stable cell line (Deli Huang, The Scripps Research Institute); Calu-3 (ATCC HTB-55), a kind gift from Dr. Catherine Chen at NCATS/NIH and Dr. Juan Carlos de la Torre at Scripps Research; Normal primary human bronchial epithelial cells (HBEC) (Lonza); HepG2 (ATCC HB-8065); HEK293T (ATCC CRL-3216)
Authentication	Cells were not authenticated.
Mycoplasma contamination	Cell lines tested negative for mycoplasma contamination.
Commonly misidentified lines<br>(See ICLAC register)	No commonly misidentified cell lines were used in this study.

Animals and other organisms

Policy information about studies involving animals; ARRIVE guidelines recommended for reporting animal research

Laboratory animals	Eight-week old male Golden Syrian hamsters, Mesocricetus auratus (Charles River).
Wild animals	The study did not involve wild animals.
Field-collected samples	The study did not involve samples collected from the field.
Ethics oversight	Hamster infection research protocol was approved and performed in accordance with Scripps Research IACUC Protocol #20-0003.<br>Pharmacokinetic studies were conducted at Scripps Research Institute's Animal Models Core in accordance with IACUC guidelines (IACUC Protocol #09-0004-5)

Note that full information on the approval of the study protocol must also be provided in the manuscript.
